# Hydrogels for Treatment of Different Degrees of Osteoarthritis

**DOI:** 10.3389/fbioe.2022.858656

**Published:** 2022-06-06

**Authors:** Shuze Wang, Yueyang Qiu, Liu Qu, Qiang Wang, Qing Zhou

**Affiliations:** ^1^ School and Hospital of Stomatology, China Medical University, Shenyang, China; ^2^ Liaoning Provincial Key Laboratory of Oral Diseases, Shenyang, China

**Keywords:** hydrogel, osteoarthritis, tissue engineering, biomaterials, bioengineering

## Abstract

Osteoarthritis (OA) is a common disease that severely restricts human activities and degrades the quality of life. Every year, millions of people worldwide are diagnosed with osteoarthritis, placing a heavy burden on society. Hydrogels, a polymeric material with good biocompatibility and biodegradability, are a novel approach for the treatment of osteoarthritis. In recent years, this approach has been widely studied with the development of materials science and tissue engineering technology. We reviewed the research progress of hydrogels in the treatment of osteoarthritis in the past 3 years. We summarized the required hydrogel properties and current applications according to the development and treatment of osteoarthritis. Furthermore, we listed the challenges of hydrogels for different types of osteoarthritis and presented prospects for future development.

## 1 Introduction

Osteoarthritis (OA) is a degenerative arthropathy that causes joint pain, limited joint motion, and joint deformity (Zhang et al., 2016). During the development of OA, the articular cartilage decomposes and thins, causing pain and swelling. As the disease progresses, the cartilage is completely worn down, and the bones scrape against each other, leading to excruciating pain, inflammation, and gradual stiffness. The number of OA patients is predicted to increase by 50% over the next two decades (Hunter et al., 2014). In the 2019 Global Burden of Disease Study, OA was the 15th largest cause of years lived with disability (YLDs) worldwide and responsible for 2% of the total global YLDs (Hunter et al., 2020).

Currently, relatively advanced treatment methods, commonly including intra-articular drug injection and cytological treatment, cannot provide satisfactory results for the treatment of OA. Intra-articular drug therapy may increase drug bioavailability and reduce the risk of systemic toxicity. However, most drug preparations used for intra-articular injection are dissolved in solution, and after injection, the drug rapidly spreads into the systemic circulation, resulting in a short retention time and a limited therapeutic effect. The cartilage layer is a dense structure filled with collagen and proteoglycans, which hinder drug penetration. In recent years, cell-based therapy has focused more on cartilage regeneration. Stem, progenitor, or primary cells are used to replace or repair OA-damaged tissue (Burke et al., 2016). However, the ability of proliferation and differentiation after implantation was reduced, and it could not provide sufficient therapeutic ability for osteoarthritis. Overall, there is no clinically convincing and effective approach to reverse the destruction of joint inflammation. Novel ideas and techniques must be used to improve the efficacy of these traditional treatments. The special physiological environment in the joint lowers the level of anabolism and proliferation of chondrocytes and weakens the ability of self-recovery.

Hydrogels comprise natural or synthetic hydrophilic polymer chains interconnected at crosslinking points. They have a unique three-dimensional network structure, material exchange ability, good biodegradability, and adjustable mechanical properties and provide a suitable microenvironment. They have considerable application prospects in tissue engineering regeneration and repair (Peppas et al., 2006). Therefore, the preparation of hydrogels with different properties as a drug release delivery system or as a cellular scaffold to promote cartilage regeneration is expected to be an effective treatment for OA.

**FIGURE 1:**
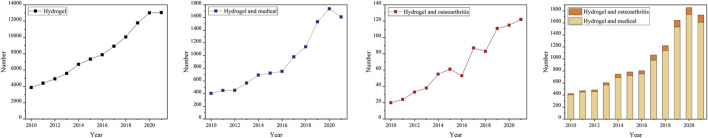
2010–2021 hydrogel-related output.

Currently, hydrogel products made from natural, synthetic, and natural–synthetic mixed-base materials have been recently developed, and an increasing number of studies focus on the use of hydrogel products in the treatment of osteoarthritis (Figure 1). In recent years, scientists all over the world have developed a variety of high-performance hydrogel products for the treatment of bone and joint inflammation. However, the special physiological environment of osteoarthritis complicates the tissue engineering treatment. Continuous exercise, a moist environment, and pressure in the joint cavity challenge the mechanical performance of the hydrogel scaffold. Simultaneously, the pathological process of osteoarthritis includes local aseptic inflammation, cartilage breakage, subchondral bone lesions, and other complex conditions. There is a significant need to develop individualized and customized hydrogel materials with different properties for different disease development and treatment methods.

In this study, clinical treatment needs were adopted as the cut-in point. First, the pathological changes of osteoarthritis and molecular mechanisms that affect the treatment process of hydrogels were reviewed and the characteristics of hydrogels required for the treatment of different degrees of osteoarthritis lesions were summarized accordingly. Finally, we emphasize the tailor-made strategy for the performance of hydrogels of different treatment methods to provide the possibility of individualized treatment of osteoarthritis, thus meeting the requirements of clinical diagnosis and treatment.

## 2 Characteristics of Hydrogel Materials Required for Treatment of Osteoarthritis

The initial pathological changes that occur at the onset of osteoarthritis are unclear; however, it is certain that various pathological changes associated with osteoarthritis are aggravated in combination, including cartilage degradation, subchondral osteosclerosis, angiogenesis, and synovitis. The difficulty of treating osteoarthritis with hydrogels is attributed to the bone’s particular internal structure and inflammatory environment, which makes both drug and cytological therapies difficult. According to previous studies, the internal environment of osteoarthritis is mainly hypoxic: the precise oxygen tension in the cartilage is estimated to be approximately 1–5%, and the gradient from the cartilage surface to the deeper area steadily decreases due to the absence of blood supply in the articular cartilage. Stem cells, which do not adapt to hypoxic conditions at the joint, cause disorders in cellular therapy for osteoarthritis (Zhou et al., 2004). Secondly, the internal environment shows abnormal stress: normal pressure is among the most important factors in maintaining chondrocyte metabolic balance (Hodgkinson et al., 2021). However, the high-stress environment caused by joint movement imposes severe stress on the mechanical properties and strength of scaffold materials. Self-healing is likewise an important property for the repair of cartilage in arthritis. Thirdly, there is lubrication in the joint cavity: the synergistic impact of synovial fluid components provides lubrication in the joint cavity, and the friction coefficient can be as low as 0.002–0.02 (Lin and Klein, 2021). Therefore, the viscoelasticity supplement is one of the important treatment methods for bone and joint inflammation.

Hydrogel materials can be adopted to treat osteoarthritis in several ways. 1) The mechanical properties of hydrogels, including pressure resistance and lubricity, are used to alleviate joint inflammation or repair the defect tissue. 2) Hydrogel can be used as the carrier of drug transportation and be implanted into the affected area by injection and open surgery, such that the utilization rate and effect of the drug can be improved. 3) Hydrogels can be used as scaffolds for cellular transport and promote the adhesion, proliferation, and differentiation of stem cells in cytological therapy.

**FIGURE 2:**
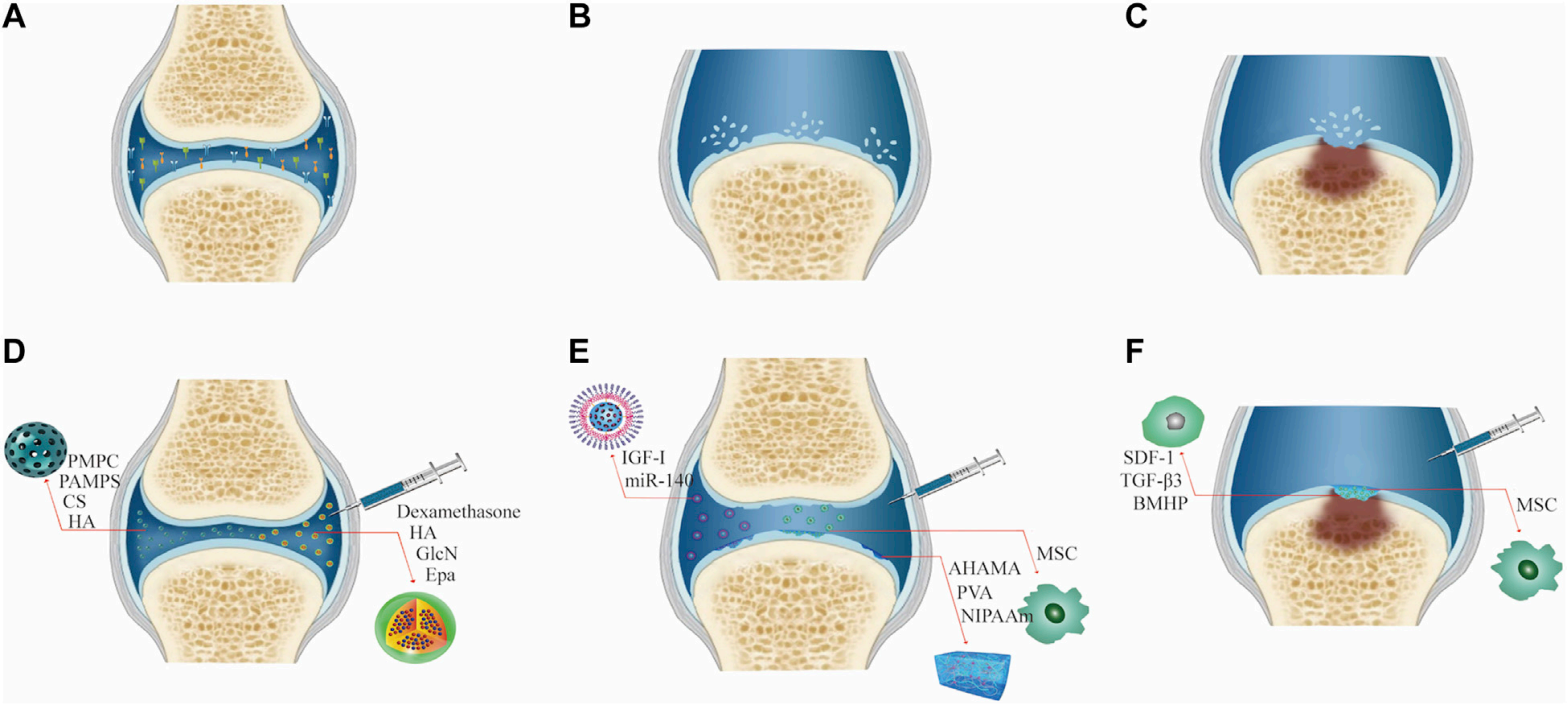
Characteristics and treatment methods of different degrees of joint OA. **(A)** Early osteoarthritis. **(B)** Advanced osteoarthritis. **(C)** End-stage osteoarthritis. **(D)** Treatment of early osteoarthritis. **(E)** Treatment of advanced osteoarthritis. **(F)** Treatment of end-stage osteoarthritis.

In terms of clinical treatment, the progress of osteoarthritis can be divided into three stages. 1) In the early stage of osteoarthritis, due to the rise in abnormal shear force in the joint cavity, the secretion of chondrocytes and the ability of proliferation are weakened. An aseptic inflammatory reaction appears in the local part of the joint cavity, resulting in the decrease of joint lubrication ability in the joint cavity. The patient experiences pain during exercise, but no evident organic pathological changes are found at this time. The elimination of local inflammatory factors, improvement of the inflammatory microenvironment, and increase in the lubrication ability of the joint cavity can significantly alleviate the joint inflammatory symptoms (Li Y. et al., 2021). 2) During the progression of osteoarthritis, the cartilage can break due to the continued existence of abnormal pressure and the continuous destruction of the inflammatory environment; however, the cartilage is not completely broken at this time, such that the subchondral bone is not exposed, which made it difficult for the stem cells in the bone marrow cavity to reach the cartilage surface for cartilage repair (Kaneshiro et al., 2006). Therefore, the treatment strategy should be mainly cytological to promote cartilage regeneration and repair. 3) At the end of osteoarthritis, the subchondral bone lesions deepen, causing subchondral bone structural disorders, including sclerosis, cystic lesions, and osteophyte formation (Burr and Gallant, 2012). It remains a major challenge to repair osteochondral lesions that involve cartilage and the underlying bone. Simultaneously, stem cells in the subchondral bone can migrate into the superior joint cavity due to the full-thickness cartilage breakage, and cell recruitment can serve as another direction for cartilage repair in osteoarthritis. (Figure 2).

## 3 Treatment Strategy of Hydrogel in Early Stage of Osteoarthritis

Studies have suggested that exploring effective treatments to prevent or slow the disease progression is of great interest for OA (Burr and Gallant, 2012). In histopathology, inflammatory cell infiltration is observed in the joint region, and soluble mediators, such as cytokines or prostaglandins, can cause chondrocytes to produce matrix metalloproteinases (MMPs) that degrade the joint synovium, triggering synovitis (Komatsu et al., 1999). Early symptoms of OA are mainly pain during joint movement and limited function. Therefore, the initial treatment strategy for joint inflammation is to mainly relieve inflammatory symptoms and improve the inflammatory microenvironment. Drugs with anti-inflammatory, lubricating, and injectable properties are often used in the treatment of OA (Berenbaum, 2013).

**FIGURE 3:**
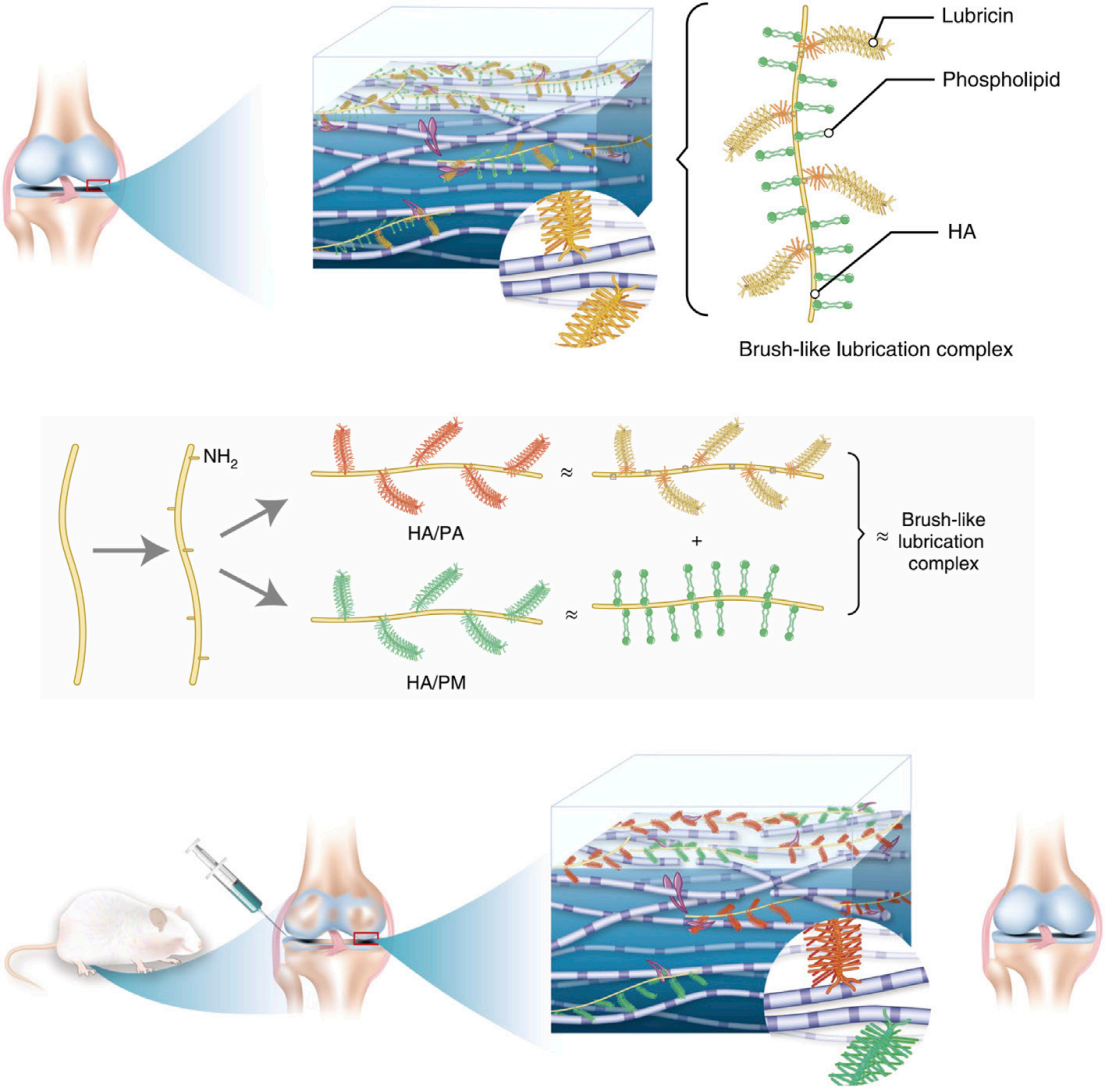
Schematic of the ideation. Illustration of the composition and structure of the lubrication complex in the normal joint. Preparation of biomimetic lubricants (HA/PA and HA/PM) that mimic the structures of the natural lubrication complex and can be used to restore the lubrication property of the joint in early OA more efficiently. Reproduced with permission from Xie R. et al., Nat Biomed Eng; published by Springer Nature, 2021.

Almost frictionless joint movements play a crucial role in daily life. The degradation of biomolecules in the synovial fluid increases cartilage wear, leading to the most common joint disease, osteoarthritis (OA) (Kosinska et al., 2015). This finding has been demonstrated in animal models of OA (Elsaid et al., 2007; Antonacci et al., 2012). The researchers believe that recovering the lubrication properties of the articular cartilage could relieve or cure OA. The nature of the hydrogel material makes it a feasible lubricant. Thus, researchers have developed various hydrogel lubrication systems based on the biomimetic boundary lubrication principle to improve the osteoarthritis microenvironment. Hydrogels based on 2-methacryloyloxyethyl phosphorylcholine (PMPC) (Milner et al., 2018), chitosan (CS) (Talaat et al., 2020), gellan gum (GG) (Leone et al., 2020), and other materials have been proposed as biomimetic lubricants to reduce cartilage damage. Xie et al. proposed a method to simulate brush-lubricated composite nanofibers (Xie et al., 2021). Two types of brush nanofibers, one of which is HA/PA, are formed by covalently grafting a high hydrophilic lubricant brush polymer poly-2-acrylamide-2-methylpropanesulfonic acid sodium salt (PAMPS) as a side chain onto the hyaluronic acid (HA) backbone (Figure 3). These biomimetic brush nanofibers form a lubricating layer on the cartilage surface and effectively lubricate the damaged human cartilage, reducing its coefficient of friction to the typical level of natural cartilage and eliminating osteoarthritis inflammation in rats over 8 weeks. Injectable lubricants with good biocompatibility may provide a novel strategy for the treatment of early osteoarthritis.

Hyaluronic acid (HA), the main component of synovial fluid (SF) in the human joint cavity (Laurent and Fraser, 1992; McDonald and Levick, 1994; Fraser et al., 1997; Scott et al., 2000), mainly acts as water and small solute flow between the articular cartilage and capillaries. However, the main disadvantage of HA is the rapid degradation in SF. Crosslinked HA hydrogels, prepared by targeting the carboxyl and hydroxyl groups of glucuronic acid or developing cysteine-derived HAs, have improved biological properties and extended retention times (Zhang et al., 2015), which compensate for their short half-life (1–2 days) disadvantage in tissues and are good viscosity supplements in the joint cavity. Mota et al. (2019) constructed an alternative delivery system on the basis of the HA hydrogel and poly (lactic-co-glycolic acid) (PLGA) particles and oleic acid. The *in vitro* release profile showed a sustained release pattern of HA from PLGA particles. The particles exhibit good *in vitro* cellular compatibility, and HA-loaded PLGA particles act as a viscous supplement and have a higher inflammatory inhibitory effect than HA solutions—indicating that the preparation may be a promising alternative to the current commercial dosage form of HA. Diaz-Rodriguez et al. (2021) combined a thermosensitive polymer (poloxamer) with hyaluronic acid to support beta-lapachone (β lap), a natural naphthoquinone, proposing it as a novel candidate for the treatment of OA. Optimized formulations were designed by the authors using artificial intelligence tools, and the hydrogel was shown to have excellent rheological properties and significantly reduced the secretion of MMP13 and CXCL8 molecules, making it a promising candidate for OA therapy.

Cai et al. (2019) synthesized a hydrogel based on HA (HA was modified by vinyl sulfone and crosslinked by dithiol-terminated polyethylene glycol). Injectable fluid hydrogels (viscous supplements) with adjustable viscoelasticity and controlled degradation rates were then prepared by adding HA-VS/SH-2-polyethylene glycol (PEG) microgels to the HA solution, exhibiting enhanced viscoelasticity, lubricating effects, triamcinolone acetonide payload capacity, high resistance to enzymatic degradation, and good biocompatibility and functionality. Furthermore, macroscopic and histological examinations of knee joints (a rabbit OA model) treated with viscosupplements indicate that the viscosupplements prevent cartilage lesions and suppress the progression of osteoarthritis. An injectable self-healing hydrogel was developed by Mou et al. (2021) . The supramolecular hydrogel exhibits good biocompatibility for chondrocytes. The intra-articular injection can significantly relieve the local inflammatory microenvironment of the knee joint by inhibiting inflammatory cytokines in SF and cartilage, such as TNF-α, IL-1β, IL-6, and IL-17. The treatment effect is better than that of HA.

As another important component of glycosaminoglycan, glucosamine (GlcN) has been extensively used to reduce joint pain and slow the progression of osteoarthritis, while the stability of polysaccharide drugs remains a major problem in the treatment. Suo et al. (2020a) prepared photocrosslinkable methacrylated gelatin (GelMA)–based hydrogels by copolymerizing GlcN acrylic acid into acryloyl glucosamine and covalently grafting it into GelMA hydrogels.

Using an injectable hydrogel sustained-release system, the efficiency of traditional osteoarthritis drugs was significantly improved, including dexamethasone, diclofenac sodium (DS), and eicosapentaenoic acid (EPA). Dexamethasone, an anti-inflammatory corticosteroid for intra-articular injection, is used in osteoarthritis treatment (Derfoul et al., 2006; Eyigor et al., 2006; Huebner et al., 2014). García-Fernández L et al. (2020) designed an injectable hydrogel-based drug delivery system (IHDDS) from oxidized dextran (Dex-ox), gelatin, and HA without using any chemical initiators. Loaded with two different anti-inflammatory drugs: naproxen and dexamethasone, the hydrogels were injected into a rabbit knee OA model. The results showed that the IHDDS was effective in alleviating the progression of OA, with a better recovery for the IHDDS-dexamethasone group. Using the characteristics of self-adhesive mussels and super-lubricated cartilage for reference, GelMA@DMA-MPC microspheres were prepared by the microfluidic technique and loaded with DS. In *in vitro* cell tests, the chondrocytes treated with interleukin-1β (IL-1β) were co-cultured with the abovementioned GelMA solutions. The results show that biocomposite microspheres significantly upregulate the expression of cartilage anabolic genes and downregulate the expression of cartilage catabolic protease genes. The benefits of the functionalized microspheres in the rat OA model were also apparent (Han et al., 2021). The injectable hydrogel microspheres provide a positive therapeutic effect on the development of osteoarthritis. Yang J. et al. (2020b) also used the microfluidic technique (MGS) to prepare microspheres and MGS@DMA-SBMA. DS is encapsulated for the treatment of OA. This mixed hydrogel has the properties of lubricity, slow degradation, and continuous drug release and has a therapeutic effect on OA. Tsubosaka et al. (2020) reported that the EPA-containing gelatin hydrogels could reduce the level of intra-articular inflammation more effectively than a single injection of eicosapentaenoic acid (EPA) in an OA mouse model, established by the surgery of destabilization of the medial meniscus (DMM). This indicates that the intra-articular injection of EPA with the hydrogel as a carrier may be a novel treatment for osteoarthritis.

Scavenging oxygen-free radicals and inhibiting the production of ROS have become important strategies for regulating inflammation in the joint cavity. Jin et al. (2020) developed an injectable hydrogel containing epigallocatechin-3-gallate (EGCG), which has intrinsic properties of modulating inflammation and scavenging free radicals. EGCG was combined with HA and gelatin to prepare the composite hydrogel, which provided protection against the proinflammatory factor IL-1β for chondrocytes. Overactivation of ASIC-3 (acid-sensitive ion channel-3) induces the expression of inflammatory cytokines in the nucleus pulposus, and APETx2 is a specific ASIC-3 inhibitor. Bian et al. (2021) constructed injectable “peptide cell hydrogel” microspheres by covalently coupling APETx2 and further loading nucleus pulposus cells. The microspheres realized controlled release of APETx2 in an acidic environment for over 28 days, which can inhibit local inflammatory cytokine storms and provide an effective method for tissue regeneration under excessive inflammatory reaction.

The active and precise release of the hydrogel material provides great convenience for the early inflammation treatment of OA. Drug delivery systems based on mechanically active biomaterials have a fine active response to mechanical signals, such as HA hydrogels with mechanically sensitive dexamethasone-loaded block copolymer micelles (BCMs) capable of continuously releasing dexamethasone in a controlled manner when the cartilage is compressed during daily activities, which can be used for pain management in patients with osteoarthritis (Lin X. et al., 2020b).

Effectively breaking through the dense structure and high-density negative charge of the cartilage matrix is key to transporting the targeted drug to the lesion area. Lin F. et al. (2021a) prepared an adhesive hydrogel microsphere with a positively charged nanometer secondary structure. The dopamine-modified structure of the microsphere surface can attach the microsphere to the cartilage surface, while the charge-guided secondary structure can carry the drug through the cartilage matrix and release them in response to reactive oxygen species. The results showed that the injectable microspheres successfully delivered antioxidants to chondrocytes in the osteoarthritis environment, which was superior to the direct drug injection control group. In recent years, the latest progress in electronics and material engineering has caused the rapid development of wireless on-demand delivery systems that use exogenous stimuli, such as acoustic waves, electric fields, magnetic fields, and electromagnetic radiation, to trigger drug carriers. Qu et al. (2018) reported that electroresponsive hydrogels prepared from the chitosan-graft-polyaniline copolymer and oxidized dextran loaded with drugs (amoxicillin/ibuprofen) show a controllable release rate set by the applied voltage. Injectable conductive hydrogels also exhibit excellent cytocompatibility and *in vivo* biocompatibility and biodegradability. Mirvakili and Langer (2021) reviewed the development of wireless on-demand delivery systems. These results suggest that wireless on-demand delivery systems could be ideal drug carrier candidates to meet the demand of chronic diseases in the future.

In summary, no cartilage defect is indicated in early arthritis, and the main therapeutic goals are to increase joint lubrication with hydrogels and thus control disease progression with their sustained release. When the cartilage defect is further developed, different repair methods must be used, depending on the depth of the defect.

## 4 Treatment Strategy of Hydrogel in Advanced Osteoarthritis

Progressive osteoarthritis is characterized by broken cartilage with partial thickness. Some of the thickness defects occur on the surface of the cartilage. The bone marrow mesenchymal stem cells (BMSCs) fail to migrate or are not recruited into the defective area (Kaneshiro et al., 2006). According to the different depth and histological features of the cartilage defect, different types of repair methods must be designed. The structure of the hydrogel can penetrate nutrients and load seed cells to strengthen the endogenous repair mechanism of damaged articular cartilage (Zhang and Khademhosseini, 2017). In clinical application, the hydrogel can be used to repair the cartilage defect in two methods: one is to load the autologous cells with the hydrogel and then implant them into the defective site (Mellati et al., 2017; Feng et al., 2019). Another method is to directly repair cartilage defects with high strength and high toughness scaffold materials. Hydrogels having one or both of the abovementioned characteristics may be suitable for cartilage regeneration. Ideal hydrogel repair materials must have the following advantages: 1) good mechanical strength and mechanical properties consistent with adjacent cartilage; 2) the fluidity of hydrogel materials that effectively mitigates irregular cartilage defects; 3) the hydrogel environment that supports cell survival and promotes stem cell proliferation and differentiation; 4) adjustable biodegradation rate; and 5) adhesion between the hydrogel and tissue (Wang et al., 2007; Zhou et al., 2018; Shirzaei Sani et al., 2019). Therefore, in addition to the lubricating and anti-inflammatory properties described earlier, this section summarizes the characteristics and application of the current hydrogel scaffolds for the advanced stage of osteoarthritis for both cytological treatment of cartilage and *in situ* cartilage repair, including their loading capacity, differentiation promoting capacity, adhesion, and mechanical strength.

### 4.1 Hydrogel and Cell Therapy

Similar to other treatment strategies, the intra-articular injection challenge serves to improve cartilage regeneration. Cell-based therapy reverses OA (Koelling and Miosge, 2009). Autologous chondrocytes and stem cells are two major cell sources for cartilage regeneration (Hoemann et al., 2005; Davatchi et al., 2011). Therefore, cell therapy for osteoarthritis is currently focused on the use of autologous chondrocytes and stem cells capable of regenerating damaged cartilage. The survival rate before and after cell transplantation is the key factor determining the therapeutic effect of stem cells. Materials studied in recent years include natural materials with good biocompatibility (HA, gelatin, alginate (Rastogi and Kandasubramanian, 2019), collagen, and the acellular matrix (Yang et al., 2018; Xia et al., 2019; Kim et al., 2021)) and synthetic materials with excellent mechanical properties and degradation rates (polyethylene glycol, poly N-isopropylacrylamide (PNIPAM), PLGA, and polycaprolactone) (Rezwan et al., 2006; Toh et al., 2011). Different material characteristics impose different therapeutic effects.

#### 4.1.1 Natural Material

Natural polymer materials have been used widely in human health engineering and regenerative tissue—for example, skin (Lin X. et al., 2021), cartilage, bone, and muscle (Lin et al., 2019)—and wound healing (Mao et al., 2020; Mao et al., 2021; Yin et al., 2021) owing to their unique advantages, such as biocompatibility, degradability, porous structure, and achievable mechanical adjustments. Although they belong to natural source materials, they have different advantages and disadvantages. These can be sorted into natural protein materials (collagen and gelatin) and natural polysaccharide materials. In addition to the advantages and disadvantages of both materials, the ability of protein materials to promote cell proliferation and differentiation is stronger.

##### 4.1.1.1 Proteins

Collagen is the most abundant protein in articular cartilage and constitutes the main component of its extracellular matrix. Collagen as a carrier of MSCs or chondrocytes has been widely studied for cartilage repair in recent years. Jiang et al. demonstrated that collagen-based hydrogels mediate the expression of Sox9, a transcription factor regulating cartilage formation, indicating that properly designed hydrogel scaffolds can act without growth factors and open novel pathways for cartilage tissue engineering (Jiang et al., 2018). However, the main disadvantage of collagen is that its mechanical properties are very unstable. High temperatures or organic solvents cause protein denaturation, which complicates the processing of collagen materials. Collagen gels tend to be too soft to contract during culture, which is detrimental to cell growth. Furthermore, the surface of the collagen gel prevents cell migration (Liao et al., 2015). Therefore, in addition to improving the processing methods of collagen, future research on modified collagen gel must focus on improving its stability, mechanical properties, and surface properties that can promote cell migration.

Gelatin, as a denatured collagen, not only has the biocompatibility and degradability of collagen but also has the adjustability of mechanical properties. Its gel state is easy to adjust and changes with the temperature (Ibusuki et al., 2003). The chemically modified HA-gelatin mixed hydrogels not only mimic the extracellular matrix (ECM) of natural tissues but also exhibit remarkable improvement in mechanical and adjustable degradation properties. Xu et al. (2019) mediated the more sustained release of small-molecule (kartogenin) and protein (TGF-β1) chondrogenic agents through the supramolecular gelatin hydrogel, which enhanced the formation of hBMSC cartilage and effectively promoted the regeneration of hyaline cartilage and subchondral bone in the rat osteochondral defect model. Gelatin methacryloyl (GelMA) is one of the hotspots in current research because it can be used as a bioink for 3D printing. The porous structure produced by 3D printing technology provides a three-dimensional culture environment for cell attachment and can produce a repair scaffold adapted to the defect site, which provides application prospects for personalized cartilage repair. Ying et al. (2020) encapsulated hMSC in a bioink based on porous gelatin methacryloyl and fabricated a micro-nano porous GelMA hydrogel structure with good injection performance by 3D biological printing, which can be used for treating cartilage defects of various shapes and sizes. Because of the layered micro-nano porous structure, the cell-loaded hydrogel structure can be easily restored to its original shape and maintain high cell viability, proliferation, diffusion, and differentiation after compression and injection. Internal studies show that the hydrogel structure can be well-integrated with surrounding host tissues. Wang K.-Y. et al. (2021a) and others synthesized modified gelatin Gel-EPL, Gel-B, GelEPL/B using ε-poly-L-lysine, and phenylboronic acid as raw materials. The gel is injectable, has good mechanical properties and biocompatibility *in vitro* and *in vivo*, and provides a good three-dimensional microenvironment for cartilage repair.

Sericin is a protein extracted from silk fiber that has high biocompatibility. One of the advantages of sericin is the nutritional supply characteristic, which allows chondrocyte proliferation even in the absence of nutrition. Sericin methacryloyl hydrogels produced by the functionalization of methacryloyl materials have similar molecular and mechanical properties to natural cartilage (Qi et al., 2018). Yodmuang et al. (2015) produced silk microfibers–silk hydrogels to further improve mechanical properties, which also produced favorable chondrocyte responses. Hong et al. (2020) developed a glycidyl methacrylated silk fibroin silk-GMA hydrogel, which, as a biological ink, demonstrated by 3D digital lighting processing printing the ability of the silk-GMA hydrogel carrying chondrocytes to reconstruct cartilage defects. However, the ability of the Silk-GMA hydrogel–loaded mesenchymal stem cells to regenerate cartilage requires further study.

As a hydrogel base material for proteins, the main problems in practical application are the difficulty of processing and the high price, which hinders its application.

##### 4.1.1.2 Polysaccharide

Polysaccharide materials have been widely studied by numerous researchers owing to their abundant reserves and stable chemical structure. Polysaccharide materials mainly include HA, chondroitin sulfate, cellulose, and alginic acid materials. The greatest advantage of polysaccharide materials is that they are highly biocompatible, have vast reserves and sources, and contain glycan components, which are similar to those of ECM. However, their shortcomings are likewise evident. Pure natural polysaccharide materials lack mechanical strength. Except for HA, they have no biological function, such as the ability to interact with proteins and cells (Lam et al., 2014). Therefore, in previous studies, multilayer materials were constructed by mixing polysaccharides with other natural materials.

HA differs from other polysaccharide materials mainly by its ability to interact with proteins and cells (Lam et al., 2014), especially when the molecular weight of HA is sufficiently high. However, as a cell delivery scaffold for the treatment of joint inflammation, HA has good biocompatibility but still needs to bind with other natural materials, especially protein materials, to undertake the role of a cell scaffold in the joint cavity. An injectable hydrogel system comprising type I collagen tyramine (Col-TA) and hyaluronan tyramine (HA-TA) was prepared as a cartilage regeneration hydrogel system loaded with BMSCs. This provides a good microenvironment for the growth and cartilage differentiation of BMSCs *in vitro* and *in vivo* (Zhang et al., 2020). Jeong et al. (2020) developed injectable supramolecular hyaluronic acid hydrogels on the basis of supramolecular chemistry between beta-cyclodextrin–modified HA (HA-CD) and adamantane-modified HA (HA-Ad) for encapsulating MSCs to control cartilage tissue regeneration. Supramolecular HA hydrogels have mechanical properties such as shear thinning, self-repair, and good cell compatibility. In cartilage defect model rats, cartilage regeneration was significant after 28 days of treatment with the supramolecular HA hydrogel encapsulating MSCs. Deng et al. (2021) studied the biofunctionalization of HA hydrogels with synthetic Wnt5a mimetic ligands (Foxy5 peptides) to promote the chondrogenesis of hMSCs. The results showed that the Foxy5 peptide–modified hydrogel encapsulated hMSCs could enhance the expression of chondrogenic markers such as SOX9, promote the metabolic activity of hMSCs, and inhibit the hypertrophy of hMSCs induced by cartilage in the hydrogel. These preliminary studies provide valuable guidance for the rational design of biologically induced scaffolds.

Alginic acid, cellulose, and chitosan-based hydrogel materials are excellent choices for environmental protection and accessibility because of their abundance in the natural environment. Alginate can be used as an injectable hydrogel for the formation of a simple gel owing to its two valence cations, such as in the calcium ion Ca^2+^. It is used as a noninvasive method for cartilage repair (Balakrishnan et al., 2014; Yan et al., 2014). Its fast-crosslinking capability is also used in 3D biometric printing (Markstedt et al., 2015; Müller et al., 2017). However, there are limitations in the single-polysaccharide materials. When the related materials are used, the physical and chemical properties can be modified or the corresponding drugs and growth factors can be added to promote the proliferation and differentiation of cells and make them more suitable for cartilage repair. Nanocellulose (NC)-chitosan (CS)/glycerophosphate (GP) thermosensitive hydrogels were prepared by Talaat et al., with biocompatibility, injectability, mechanical stability, and slow degradation. Human dental pulp stem cells (hDPSCs) embedded in NC-CS/GP hydrogels showed cartilage differentiation ability *in vivo* (Talaat et al., 2020). Zhang et al. prepared triple interpenetrating network hydrogels (comprising dextran, chitosan, and teleostean) loaded with mesenchymal stem cells (MSCs) for the treatment of intervertebral disk degeneration in a goat model (Zhang C. et al., 2021). The results showed that the hydrogels improved the intervertebral disk height index and histology condition. These encouraging findings inspire further long-term studies of the therapeutic effects of hydrogels and MSC injections in this large animal model.

##### 4.1.1.3 DNA Hydrogel

The unique characteristics of DNA hydrogels, such as sequence programmability, accurate molecular recognition, stimulation responsiveness, biocompatibility, and biodegradability, are useful in applications ranging from material science to biomedical applications (Basu et al., 2020; Yao et al., 2021). DNA supramolecular hydrogels were used to deliver MSCs for the treatment of severe OA. Researchers systematically investigated the effect of the DNA supramolecular hydrogel on delivered cells both *in vitro* and *in vivo* (Yan et al., 2021). A harsher friction OA environment was built in a rabbit OA model, which was established by simultaneous anterior cruciate ligament transection and complete medial meniscectomy (MMx). The experimental results demonstrated that DNA supramolecular hydrogels could promote cartilage formation, reduce bone proliferation, and normalize subchondral bone under high friction conditions of OA. Thus, DNA supramolecular hydrogels may be used as an effective cell delivery system for mesenchymal stem cell therapy.

#### 4.1.2 Synthetic Hydrogel

Synthetic hydrogels with a controllable three-dimensional structure and adjustable mechanical strength can be loaded with cells for cartilage regeneration. In particular, the application and development of hydrogels based on PEG have become a research hotspot. This material was approved by the Food and Drug Administration for medical applications and personal care products. However, the common problem of bioinertia in PEG persists.

The use of copolymers to improve cell adhesion and cartilage formation of PEG materials has demonstrated its efficiency. The transition temperature, pore size, and mechanical stress of the triblock copolymer can be adjusted in a wide range. Simultaneously, this shows great potential in promoting cartilage regeneration by loading stem cells and drugs or biological molecules that promote cell differentiation. In the study of Li et al. (2020), gellan gum (GG)/polyethylene glycol acrylate (PEGDA) double-network (DN) hydrogels were prepared by combining GG with PEGDA, which exhibited excellent mechanical and relaxation properties, similar to the microenvironment in natural ECMs, thus guiding the behavior of stem cells. *In vivo* implantation of DN hydrogels containing cartilage-inducing factors indicates that GG/PEGDA DN hydrogels enhance cartilage differentiation of BMSCs and have attractive prospects in the application of cartilage tissue engineering. Wu et al. (2021) developed a series of injectable viscoelastic polyethyleneglyceride (PEGS-OH) hydrogels. By changing the composition and crosslinking degree, it was found that 80% crosslinked PEGS-OH promoted the adhesion and differentiation of bone mesenchymal stem cells significantly higher than the 100% and 60% crosslinking degrees and resulted in faster cell stress release, which was beneficial to the adhesion and differentiation of BMSCs. These results provide design guidance for the next generation of biomaterials.

#### 4.1.3 Hybrid Hydrogel

Both pure natural hydrogel and synthetic hydrogel materials have difficulty meeting the requirements of ideal scaffold materials. Therefore, it is an important approach to combine synthetic and natural materials by physical methods or chemical crosslinking to form hybrid hydrogels in the field of cartilage repair. Another important research direction of the PEG material is as a high-quality additive material in the cell-loaded hydrogel, which increases the mechanical strength of the hydrogel material and is more widely used by combining with the natural material to improve its biological function. For example, Cho et al. (2020) developed an implantable dual delivery platform in thiolated gelatin (gelatin-SH)/poly (ethylene glycol) diacrylate (PEGDA) interpenetrating network (IPN) hydrogels with coacervates (Coa), a tertiary complex of poly (ethylene argininylaspartate diglyceride) (PEAD) polycation, heparin, and cargo insulin-like growth factor-1 (IGF)-1. Gelatin-SH/PEGDA IPN hydrogels exhibit biocompatibility and mechanical properties and can be used for long-term transplantation. PEAD-based Coa sustainably releases bioactive IGF-1 within 3 weeks. By enhancing the deposition of glycosaminoglycan and the expression of chondrogenesis-related genes, the quality of the regenerated tissue in the cartilage layer and the subchondral layer of the rabbit full-thickness osteochondral defect model was significantly improved 12 weeks after implantation. PEGylated poly (glycerol sebacate) (PEGS) scaffolds with controlled degrees of crosslinking and gradable macroscopic and microscopic porosity were prepared by solvent-free urethane crosslinking and spontaneous pore formation at room temperature (Lin D. et al., 2020). Compared to three different crosslinking durations (12, 24, and 48 h), PEGS-12 h with a lower degree of crosslinking significantly stimulated chondrocyte differentiation and maintained the chondrocyte phenotype and better-enhanced cartilage matrix secretion. On this basis, a dual-function PEGS/MBG double-layer scaffold was constructed by combining PEGS-12 h with a mesoporous bioactive glass (MBG) to successfully reconstruct the intact articular hyaline cartilage and its subchondral bone.

Liu et al. (2021) designed a 3D biologically printed multilayer scaffold containing MeHA/polycaprolactone, combined with kartogenin (KGN) and β-tricalcium phosphate (β-TCP), which showed that BMSCs in the scaffold survived and proliferated *in vitro* and produced a significant amount of cartilage-specific extracellular matrix. In an animal model of OA induced by medial meniscectomy, the stent promotes cartilage formation by promoting type II collagen synthesis in cartilage defects and inhibiting interleukin-1 beta. Li P. et al. (2021) developed a cartilage regeneration system on the basis of a chitosan hydrogel/3D printed poly-ε- caprolactone mixture that contains synovial stem cells (SMSCs) and recruits tetrahedral framework nucleic acids for injection into the joint cavity. Three-dimensional printed PCLs provide basic mechanical support, and TFNAs provide a good microenvironment for the proliferation and cartilage differentiation of transported SMSCs and promote cartilage regeneration. The self-assembled polypeptide hydrogel was coated on the surface of the PCL fiber and filled into the interstitial space of the scaffold to construct the SAPH-PCL composite scaffold (Li et al., 2019). The structure of the composite scaffold is similar to that of the extracellular matrix (ECM). The composite scaffold effectively avoids excessive loss of nutrients, maintains the three-dimensional microenvironment of cell growth, promotes the layered deposition of the cartilage and osteogenic matrixes, and reconstructs the microenvironment of the osteochondral defect area. In summary, scaffold-loaded stem cells are a promising strategy for cartilage regeneration in combination with enhanced cell proliferation and cartilage formation.

In addition to the abovementioned applications, we believe that the most promising cytological treatment scaffold for articular cartilage injury must be a biomimetic natural cartilage scaffold. Zhang H. et al. (2021) designed a 3D-printed gradient hydrogel scaffold of similar structure to osteochondral tissue, including pure hydrogel–based cartilage in the top layer, calcified cartilage in the middle layer containing 40% nano-hydroxyapatite (nHA) and 60% hydrogel, and a 70/30% nHA/hydrogel in the bottom subchondral bone layer. BMSCs loaded with gradient scaffolds showed better repair than other scaffolds. Qiao et al. (2021) designed and manufactured three-layered scaffolds where mesenchymal stem cell (MSC)-gelatin methacrylamide (GelMA) hydrogels rich in region-specific growth factor delivery were combined with molten electro-triblock polymers of poly (ε-caprolactone) and poly (ethylene glycol) (PCEC) networks and deeply dependent fibrous tissue. The introduction of PCEC fibers into weak gel hydrogels contributes to the improvement of mechanical strength. The system combines rigid and flexible components as a filler for local cartilage defects and can be used to develop layered multitissue scaffolds. *In vitro* biological experiments showed that layered fiber-reinforced and growth factor–loaded hydrogel structures induced MSC differentiation, forming cartilage and osteogenic lineage cell phenotypes that are similar to natural tissue matrix accumulation. Simultaneous cartilage and subchondral bone regeneration enable the use of three layers of integrated scaffolds *in vivo*. Biologically inspired constructs that mimic spatial changes in natural osteochondral tissue may be promising candidates for enhanced osteochondral regeneration.

Xing et al. (2021) constructed a double-layer hydrogel with gellan gum/alginate (GG/ALG) in the upper layer and gellan gum/hydroxyapatite (GG/HAp) in the lower layer, which mimics the mechanical properties and interface structure of the gradient in the osteochondral tissue, can induce BMSCs to differentiate into cartilage and osteocytes *in vitro*, and can repair the osteochondral defects in rabbit femoral intercondylar osteochondral defects *in vivo*. Chen Z. et al. (2021) manufactured a heterogenous double-layer hydrogel scaffold. Gelatin GelMA and acryloylglucosamine are the main components in the upper layer, simulating the cartilage ECM. At the same time, vinylphosphonic acid (VPA), as a non-collagen analog, is incorporated into the bottom layer to induce *in situ* biomineralization of calcium phosphate. The two heterogeneous layers are effectively sewn together by the chelation of calcium ions and alginate added to the upper and lower layers. This cell-free, economical, and efficient hydrogel has shown great potential in osteochondral repair and inspired the design of other tissue engineering scaffolds.

Gene vector delivery guided by the hydrogel material is a new and attractive therapeutic solution for targeted cartilage repair. Gene vectors can be precisely controlled and delivered in a minimally invasive and spatiotemporal manner to reduce the dispersion of intra-articular vectors and the possible loss of therapeutic gene products. The Magali Cucchiarini team (Madry et al., 2020) used an injectable and thermosensitive hydrogel on the basis of polyethylene oxide (PEO)–polypropylene oxide (PPO)–PEO-poloxamer to control the release of therapeutic recombinant adeno-associated virus (rAAV) vectors that overexpress chondrogenic Sox9 transcription factors in full-thickness cartilage defects, significantly improving cartilage repair, bringing collagen fiber orientation closer to normal cartilage, and protecting subchondral bone plates from early bone loss. Maihöfer et al. (2021) and others delivered the alginate hydrogel (IGF-I/AlgPH155) to recombinant human adeno-associated virus (rAAV) vector encoding human insulin-like growth factor I to improve full-thickness cartilage repair. In the cartilage defects after fracture in minipigs, sustained insulin-like growth factor-1 (IGF-1) overexpression was significantly realized in the cartilage surrounding the defects. The cartilage repair parameters were significantly improved without harmful or immune responses; they significantly reduced perifocal OA and inflammation. Biomaterial-guided rAAV gene transfer represents a valuable clinical method to promote cartilage repair and prevent OA. Li et al. (2022) used a multifunctional gene vector, arginine, histidine, and phenylalanine-modified fifth-generation polyamides (designated G5-AHP) to form G5-AHP/miR-140 nanoparticles by forming a complex with microRNA-140 (miR-140). Nanoparticles were encapsulated in hydrogel microspheres (MSs) to construct “nanometer-micron” composite gene hydrogels to reduce the degradation of articular cartilage. G5-AHP/miR-140 nanoparticles released from MSs showed high gene transfection efficiency and long-term biological activity, promoting endocytosis and thereby maintaining the metabolic balance of the cartilage matrix by promoting the expression of type II collagen and inhibiting the expression of cartilage matrix. These studies provide a new cell-free method to alleviate the progression of OA, which suggests the potential of locally injected gene delivery systems.

### 4.2 *In Situ* Hydrogel Repair

In the repair of cartilage defects, direct scaffold material filling with *in situ* damage has a high requirement for physical and chemical properties and cell compatibility of the scaffold material. Due to the movement function of the joint, fatigue resistance, mechanical properties, and adhesion to the surrounding tissue of the scaffold, the material has high requirements. As the main component of natural cartilage, chondroitin sulfate and fibrin-based hydrogel materials can form a good three-dimensional network structure. Such hydrogel materials, however, can be cleaved by enzymes in surrounding tissues, making it difficult to maintain stability. In the study by Weizel, the mechanical behavior of human cartilage, chondro filler liquid, and ADA-GEL under various loading modes was characterized, also *via* cyclic loading and stress relaxation experiments. The results show that the materials exhibit different properties in compression-tensile asymmetry, nonlinearity, recoverability, adjustment, and stress relaxation, which is consistent with the differences in the material microstructure (Weizel et al., 2020).

The most prominent advantage of the gelatin-based hydrogel material is its adjustable tissue adhesion, which has a great influence on the stability and repair of the material after implantation and makes it relatively stable in complex joint movement. Chen J. et al. (2021) prepared the injectable binder hyaluronic acid hydrogel modified with the aldehyde group and methacrylate (AHAMA) on a polysaccharide skeleton, which has various anchoring mechanisms (formation of amide bonds by dynamic Schiff base reaction, hydrogen bonds, and physical interpenetration). Compared with commercial fibrin glues (∼10 kPa) and HAMA hydrogels (∼20 kPa), AHAMA hydrogels exhibit significantly improved durability and stability in wet environments (at least 7 days) and higher adhesion strength (43 kPa for skin and 52 kPa for glass). More recently, a combination of photopolymerizable gels characterized the photopolymerizable HA and allogeneic acellular cartilage matrix (DCM) (Wang Y. et al., 2021). DCMs harvested from abnormal pigs with the α-1, α-1-galactose gene knockout suggests that DCMs play an important cartilage adhesion–enhancing role. This is potentially useful in regenerative medicine.

Xia et al. (2018) combined 3D printing, photo-crosslinking, and lyophilization to prepare hydrogels based on gelatin and HA and used 3D printing to ensure the precise control of external 3D shapes and internal pore structures. Lyophilization was used to further improve mechanical properties and prolong degradation time. The scaffolds obtained an appropriate internal pore structure suitable for cell distribution, adhesion, and proliferation. *In vitro*, cartilage-like tissues were gradually formed in the scaffolds after chondrocyte seeding. *In vivo*, the chondrocyte-scaffold was subcutaneously implanted into an autologous goat model and successfully regenerated mature cartilage with a typical lacunae structure and cartilage-specific extracellular matrix. Although the application of scaffolds in OA treatments must be further explored *in vitro* and *in vivo*, the current study provides a promising strategy for the repair of cartilage defects.

**FIGURE 4:**
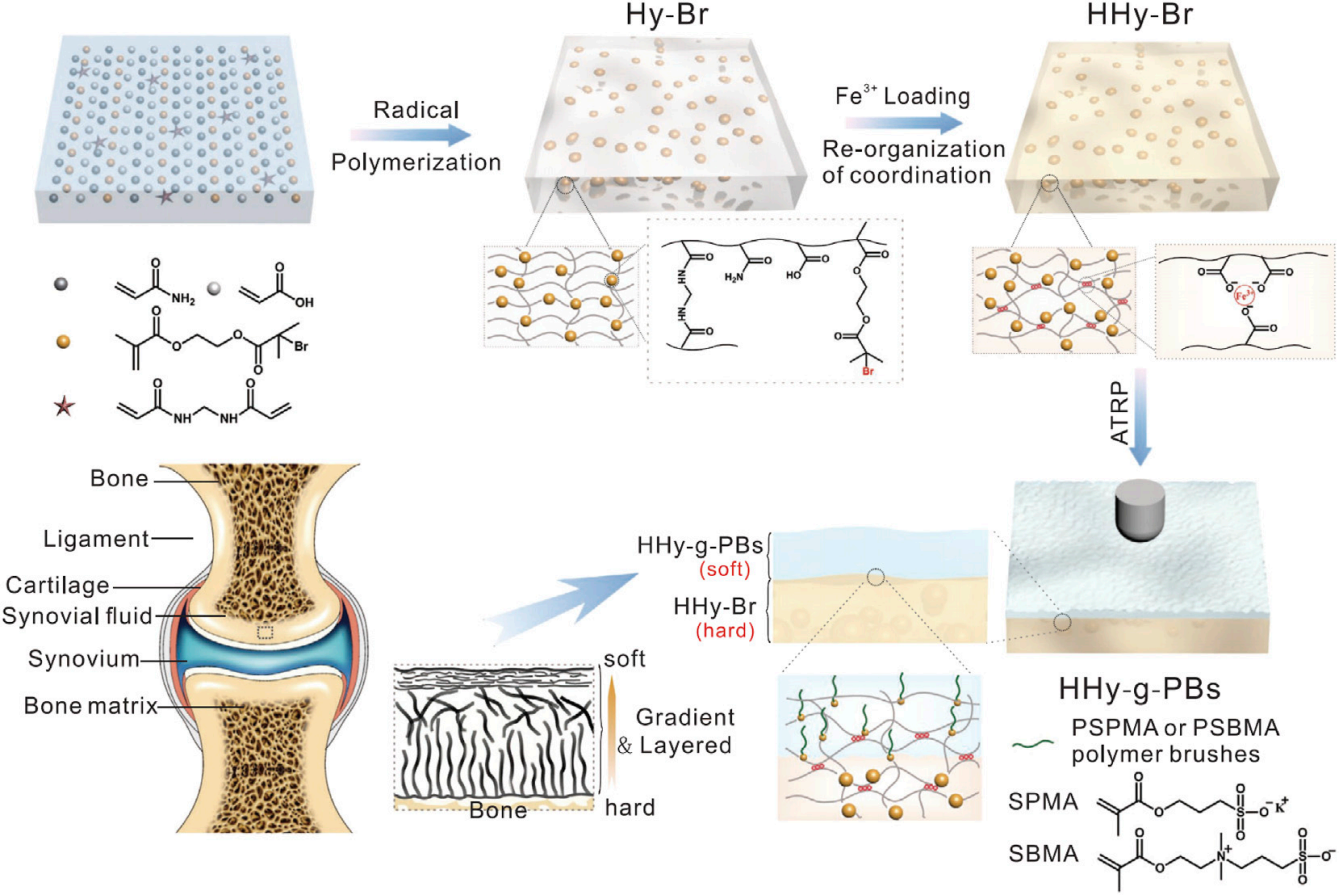
Schematic of cartilage-like hydrogel. Illustration of fabrication procedures of PSPMA or PSBMA brush-grafted hydrogels. The high-strength hydrogel substrate with embedded BrMA was fabricated by radical polymerization and post-treatment with ferric ions. Subsequently, the layer of polymer brush–grafted hydrogel (HHy-g-PBs) was prepared by the ATRP method at the subsurface. Combining the soft HHy-g-PBs layer with a strong HHy-Br substrate could mimic the structure of articular cartilage. Reproduced with permission from Rong M. et al., *Adv Funct Mater*; published by Wiley-VCH, 2020.

The improved mechanical properties expand the application of GelMA material, especially in the process of arthroscopic combined treatment, and sufficient mechanical strength can cooperate with arthroscopic operation, directly introducing the cartilage defect under the direction of arthroscopy. This improves the accuracy of injection, thereby improving the accuracy and speed of treatment. The light source may be shared with an arthroscopic instrument, and the transition from the injected liquid to the hydrogel may be achieved *in situ* by thermal activation (when the hydrogel is crosslinked at body temperature) or by light crosslinking using ultraviolet light (e.g., with gelatin methacrylamide or HA). Gang, 2022 showed a simple and effective strategy for preparing hydrogels with high strength, high toughness, rapid self-recovery, and cell compatibility. Hydrogels were prepared using nano-Fe_3_O_4_ as a physical crosslinking agent based on the dual network structure of the flexible AAm binary polymer and rigid chitosan crosslinking. Taking full advantage of the synergistic effect of the DN strategy and weak non-covalent interaction (including metal coordination and hydrogen bond) can effectively improve the strength, toughness, and self-recovery properties of composite hydrogels. With the help of the strategy, the originally weak hydrogels are more promising in the field of osteochondral repair. Rong et al. (2020) reported a double-layer hydrogel material that mimics natural cartilage. The material chemically inserts a hydrophilic polyanion PSPMA brush or polyzwitterion PSBMA brush into the surface of a high-strength hydrogel using an initiator to form a robust polymer brush graft hydrogel composite (Figure 4). Their synergistic effect could achieve a low coefficient of friction (grade 0.010) under heavy underwater loading conditions (contact pressure class 10 MPa), with a performance similar to that of natural articular cartilage. Moreover, even when subjected to 50,000 reciprocating cycles at high contact pressures, the hydrogel can maintain low friction, and almost no wear is observed. This research opens up a new technological path for the development of ultra-low friction soft materials for biomimetic cartilage.

Poly (vinyl alcohol) (PVA) hydrogel is a reliable and high-performance carrier (Lu et al., 2017). PVA hydrogels formed by casting drying methods have been reported to have similar mechanical properties to natural human cartilage, indicating that such hydrogels can be used for cartilage replacement (Oliveira et al., 2019). Yang F. et al. (2020b) reported the first hydrogel with tensile and compressive strength and modulus of cartilage and the first hydrogel with equivalent tensile fatigue strength of cartilage at 100,000 cycles. These properties are realized by infiltrating the bacterial cellulose nanofiber network with a poly (vinyl alcohol) (PVA)-poly (2-acrylamido-2-methyl-1-propanesulfonic acid sodium salt) (PAMPS) double-network hydrogel. The BC provides tensile strength in a collagen-like manner in cartilage, while PAMPS provides a fixed negative charge and osmotic resilience similar to the action of polymeric proteins in the cartilage. The hydrogel has the same aggregation modulus and permeability as the cartilage, causing the same time-dependent deformation under limited compression. The hydrogel’s properties make it an excellent candidate for replacing damaged cartilage. Lan et al. (2020) prepared PVA/Col-II chondrohydrogels with high interconnection, porosity structure, moderate elastic modulus, and good biocompatibility using the freeze–thaw method. The 11 ± 1.7 kPa of PVA/Col-II 1:1 is significantly higher than that of 4.9 ± 0.6 kPa. In addition, all porous hydrogels have good cell compatibility. The CCK-8 results showed that the cell proliferation rate of PVA/Col-II 1: 1 hydrogel was higher than that of other hydrogels.

Numerous research teams have chosen PEG as the base material to design hydrogels for cartilage repair, mainly owing to its good mechanical properties and ease of modification (Lin, 2015). In particular, the layered design for cartilage defect repair provides the hydrogel materials with different performance characteristics with their own strengths and physicochemical properties. Means et al. (2019) developed a dual-network hydrogel consisting of a first network poly (2-acrylamide-2- methylpropanesulfonic acid) (PAMPS) and a second poly (N-isopropylacrylamide-co-acrylamide) [P (NIPAAm-co-AAm)], which was evaluated as a potential ready-made material for cartilage replacement. The compressive strength of these PNIPAAm-based DNs (approximately 25 MPa, similar to cartilage) was increased by a factor of 50 compared to conventional single-network hydrogels, while cartilage-like modulus (about 1 MPa) and hydration (∼80%) were achieved. Compared with healthy cartilage (pigs), these hydrogels are not only parallel to the strength, modulus, and hydration of the natural articular cartilage but also have a 50% lower coefficient of friction. The special cartilage characteristics of PAMPS/P (NIPAAm-co-AAm) DN hydrogels make it a candidate for cartilage defect repair of synthetic cartilage grafts. Therefore, natural biological macromolecules can bind to synthetic polymers and have good mechanical strength and biological function.

It is difficult for hydrogel materials to retain their adhesive ability in moist environments. The limited volume of the joint cavity and excessively large or complex molecular polymers make the material of excessive size and lose its application value in clinical treatment. With the further study of marine organisms, researchers have discovered the law of underwater adhesion of mussels and developed a series of high strength, high adhesion, and high biocompatibility hydrogel materials for the joint cavity environment. For example, Han et al. (2018) designed a polydopamine-chondroitin sulfate polyacrylamide (PDA-CS-PAM) hydrogel with tissue adhesion and ultra-mechanical properties for cartilage regeneration without growth factors. Due to the abundance of reactive catechol groups on PDAs, PDAs and CSs are self-assembled to form cartilage-specific PDA–CS complexes, which are then uniformly bound to an elastic hydrogel network. This catechol-rich PDA–CS complex imparts good cell affinity and tissue adhesion to hydrogels to promote cell adhesion and tissue integration. In general, this tissue-adhering and tough PDA-CS-PAM hydrogel with good cell affinity creates a growth factor–free biomimetic microenvironment for chondrocyte growth and cartilage regeneration and provides inspiration for the development of growth factor–free biomaterials for cartilage repair.

To mimic the structural characteristics of articular cartilage with a natural cell gradient from tissue surface (high cell) to depth (low cell), researchers used magnetically controlled methods to construct hydrogels with different cell gradient densities (Zlotnick et al., 2020). Magnetically controlled methods have broad prospects in generating cell gradients, especially in the construction of bone and cartilage tissues. In the future, we can attempt to construct the gradient tissue with different magnetic chondrocytes and bone-stimulating factors and expect to produce continuous cartilage and bone tissue.

## 5 End Treatment of Osteoarthritis

The full-thickness defect implies that the cartilage defect is deep and penetrates the tidal line but does not penetrate the subchondral bone. An osteochondral defect indicates that the defect is deep enough to reach the bone marrow. Full-thickness defects and osteochondral defects can be partially repaired by bone marrow stromal cells (BMSCs) (Hunziker, 2002; Zhang et al., 2013). The end-stage treatment of osteoarthritis is characterized by the exposure of the subchondral bone due to full-thickness destruction of the articular cartilage compared to the advanced stage. The stress of the subchondral bone in the articular cavity changes, and the sclerosis of the subchondral bone and the formation of the osteophyte make it necessary to repair the bone tissue. The stability of composite cells after implantation of scaffold materials is likewise more challenging. Simultaneously, stem cells in the subchondral bone can migrate into the superior joint cavity due to the full-thickness cartilage breakage, and cell recruitment can serve as another direction for cartilage repair in osteoarthritis. Two main approaches to improve cell recruitment are to increase the concentration of chemokines in the damaged site and increase the number of stem cells in the damaged local microenvironment. Stem cells can be recruited either directly from stem cell pools that damage the surrounding tissue or from the circulatory system. Because there is no need to culture and expand endogenous stem/progenitor cells *in vitro*, there is no risk of immunogenicity and disease transmission. The hydrogel base material may provide conditions for cell recruitment. In addition, the mechanical properties of the cartilage surface and osteochondral tissue are different. It may be better to repair osteochondral defects with multilayered scaffolds with graded tissue.

**FIGURE 5:**
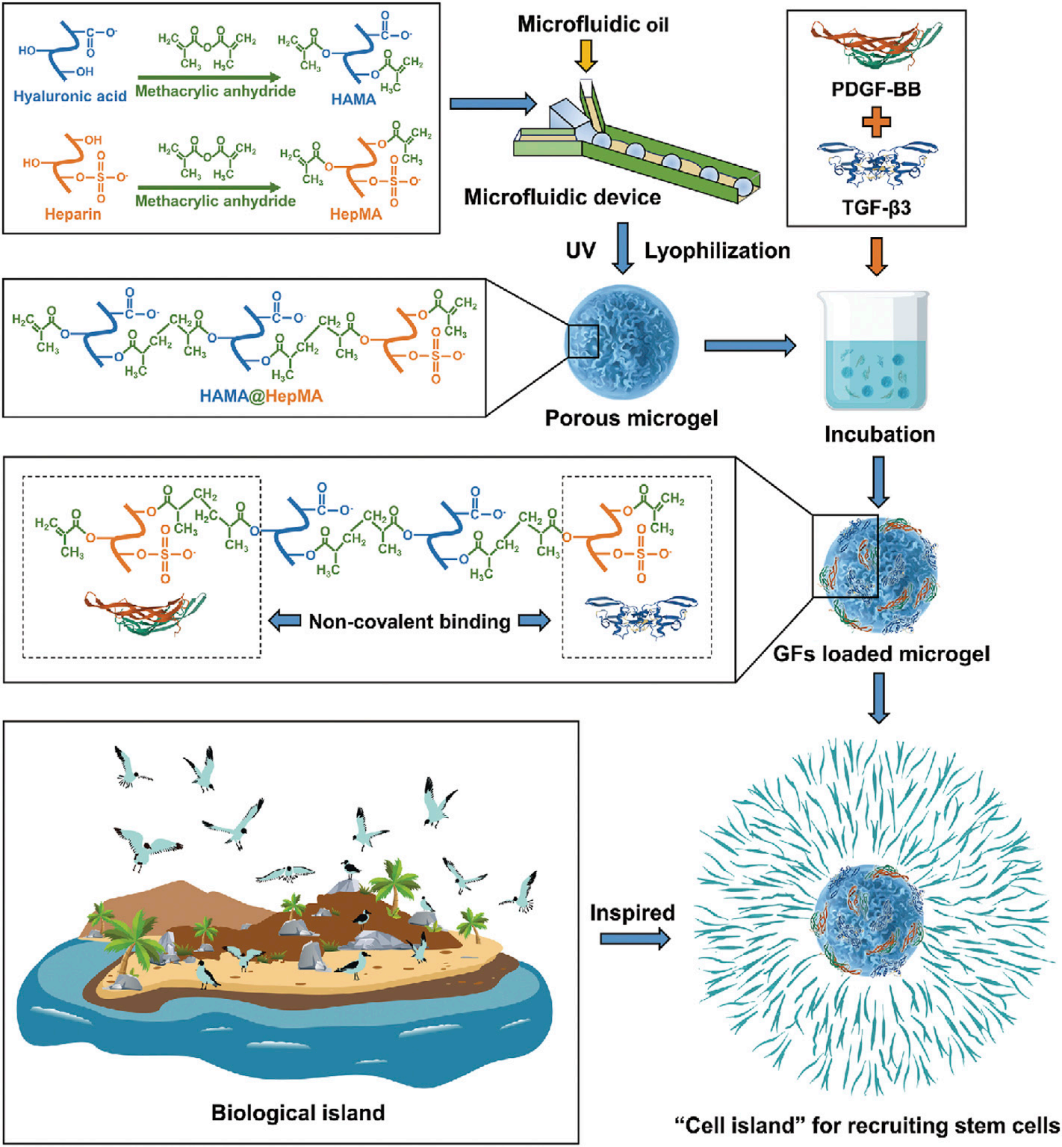
Illustration of the fabrication of “cell island” microgels. The injectable porous microgel was fabricated by photopolymerization of methacrylated hyaluronic acid and heparin (HAMA@HepMA) blend pregel droplets generated *via* microfluidic technology. Subsequently, PDGF-BB and TGF-β3 were non-covalently incorporated within the microgels by binding heparin, forming “cell island” microgels that can directly recruit stem cells post injection. Reproduced with permission from Lei Y. et al., *Adv Funct Mater*; published by Wiley-VCH, 2021.

Lu et al. (2018) combined the directed acellular cartilage matrix (ACM) with bone marrow homing peptide (BMHP) functional self-assembled peptide to construct a composite hydrogel scaffold. Dong et al. (2021) developed a cellular cartilage tissue engineering system using injectable chitosan/silk fibroin hydrogels. The hydrogel system can release stromal cell–derived factor-1 (SDF-1) first and then kartogenin (KGN) in a unique sequential drug release pattern, which can promote mesenchymal stem cell recruitment and cartilage differentiation in time and space. The hydrogel has good injectability and reticular porous structure. The microspheres were uniformly distributed in the hydrogel and allowed the sequential release of SDF-1 and KGN. The results showed that the hydrogel system had good cell compatibility and promoted the migration and differentiation of MSCs into chondrocytes. *In vivo* experiments of rabbit articular cartilage defects showed that the cell-free hydrogel system was beneficial to cartilage regeneration. Thus, the composite hydrogel system shows potential for application in cellular cartilage tissue engineering. In the study by Lei et al. (2021), injectable methacrylated hyaluronic acid and heparin (HAMA@HepMA) blend MGs were prepared by combining microfluidic technology and photopolymerization processes. Platelet-derived growth factor-BB (PDGF-BB) and transforming growth factor-beta3 (TGF-β3) (GFs) were non-covalently incorporated within the MGs by binding heparin, forming “cell island” MGs that can not only recruit endogenous stem cells but also promote chondrogenic differentiation. Based on the abovementioned advantages, the “cell island” MGs might be used in the treatment of osteoarthritic cartilage damage (Figure 5).

Zhang F.-X. et al. (2021b) developed an exosome-loaded high-adhesion hydrogel for endogenous cell recruitment and cartilage defect regeneration using the crosslinking network of sodium alginate–dopamine, chondroitin sulfate, and regenerated silk fibroin (AD/CS/RSF). Compared to commercially available enbucrilate tissue adhesives, AD/CS/RSF hydrogels have similar gelation times and stronger adhesiveness. The AD/CS/RSF/EXO hydrogel containing exosomes can promote the migration and expansion of endogenous BMSCs, promote the proliferation and differentiation of BMSCs, and accelerate the regeneration of *in situ* cartilage defects and the remodeling of the extracellular matrix. HUC-MSCs-sEVs derived from human umbilical cord mesenchymal stem cells promote the migration, proliferation, and differentiation of chondrocytes and hBMSCs. Hu et al. (2020) prepared Gelma/Gel-nano hydrogels for the sustained release of sEVs. *In vivo*, GelMA/Gel-nano-sEVs hydrogels containing hUC-MSCs-sEVs could effectively promote cartilage regeneration. These results suggest that Gel-nano-sEVs have a good ability to stimulate cartilage formation and repair cartilage defects.

For severe osteoarthritis with cartilage defects, stem cell therapies have obtained effective results in the laboratory for both exogenous and autologous stem cell recruitment (Zhang et al., 2020). However, compared to autologous stem cell recruitment, the limitations of exogenous stem cell transplantation are emerging. The limited availability of stem cell sources, low viability, technical complexity, high medical costs, and ethical and safety issues in the clinical translation are evident drawbacks of stem cell transplantation (Zhang et al., 2019). The limitations can be summarized in three approaches. 1) The acquisition of stem cell acquisition. Stem cells for articular cartilage repair usually originate from specific locations in adults, such as adipose tissue, bone marrow, and synovium. The largest advantage of adipose-derived stem cells (ADSCs) is that they are easily collected by surgical procedures (Pizzute et al., 2015). This solves numerous problems and safety and ethical issues. Furthermore, compared with other stem cells, the efficiency of extraction for ADSCs is higher (Bajek et al., 2016). However, due to the ability of ADSCs to repair and because the level of cartilage differentiation is not as good as in synovium-derived stem cells (SMSCs), it is not the first choice for articular cartilage repair. Bone marrow–derived mesenchymal stem cells (BMSCs) are widely used in cartilage regeneration studies, whereas the recognized source of stem cells for cartilage repair has not been found. Synovium-derived stem cells (SMSCs) have good cartilage differentiation potential, possibly due to the high expression of proline/arginine-rich end leucine-rich repeat protein, which is glycoprotein rich in cartilage but has little or no content in stem cells outside the joints (Pizzute et al., 2015). However, its acquisition is often accompanied by damage to joint tissue. Moreover, the quantity of synovial tissue is small, especially in smaller joints, such as temporomandibular joints, where it is difficult to collect sufficient amounts of SMSCs. 2) Mode of stem cell delivery. The method by which stem cells are transported affects the success rate of stem cell transplantation to a certain extent. It is impossible to accurately target the area of cartilage after the direct injection of stem cells into the articular cavity. Therefore, it is of great interest to effectively locate stem cells to the site of injury and enhance their chondrogenesis. 3) Effects after stem cell implantation. There are several risks of exogenous stem cell implantation, such as the risk of tumorigenesis, disease transmission, and immune rejection (Večerić-Haler et al., 2017). Furthermore, the influence of the osteoarthritis microenvironment on the differentiation ability of stem cells remains a research hotspot.

The abovementioned limitations can be improved by using autologous stem cell recruitment, and we have reason to believe that tissue engineering technologies with strong stem cell recruitment capability are highly promising in cartilage repair.

## 6 Challenges

To date, the performance of scaffold materials for the treatment of OA is still being breaken through and improved constantly. The physiological environment of osteoarthritis makes the requirements of tissue engineering materials very complex and requires a variety of performance superpositions, even though some performance is contradictory. Materials require strong mechanical strength, controlled absorption efficiency, good biocompatibility, drug or cell loading capacity, and bone or chondrogenesis. Furthermore, in recent years, tissue adhesion, anti-fatigue performance, and self-healing performance have been found to exert great influence on the treatment effect of arthritis. Moreover, the required hydrogel stent performance varies for different osteoarthritis types. Hydrogel scaffolds with different substrates exhibit different performance characteristics. How to fuse different materials and obtain their common advantages is an urgent problem. Machine learning and artificial intelligence can help us simulate material hybridizations, reduce the number of our experiments, replace our trial and error, and potentially help us find the scaffold material that is ultimately suitable for osteoarthritis treatment.

The development process and treatment of osteoarthritis are highly complicated, which brings significant trouble to doctors and patients in clinical diagnosis and treatment. The main reason is that a bone-cartilage unit consisting of subchondral bone and cartilage plays an important role in joint homeostasis and OA development. At the same time, two different joint units experience different abnormal changes in terms of their tissue structure and cell activity. However, the current research and development of biomaterials and related therapies are mostly aimed at a single structural unit, which may significantly limit the therapeutic effect. The cross-talk between chondrocytes and subchondral osteocytes and the interaction of the microenvironment in the pathological state have not been elucidated in the pathophysiology. Therefore future research should focus on the pathological processes of cartilage and subchondral bone, the relationship between cell interactions and OA progression, as well as the development of targeted biomaterials for simultaneous treatment of compound joint units to achieve better therapeutic outcomes.

## 7 Conclusion and Prospects

**FIGURE 6:**
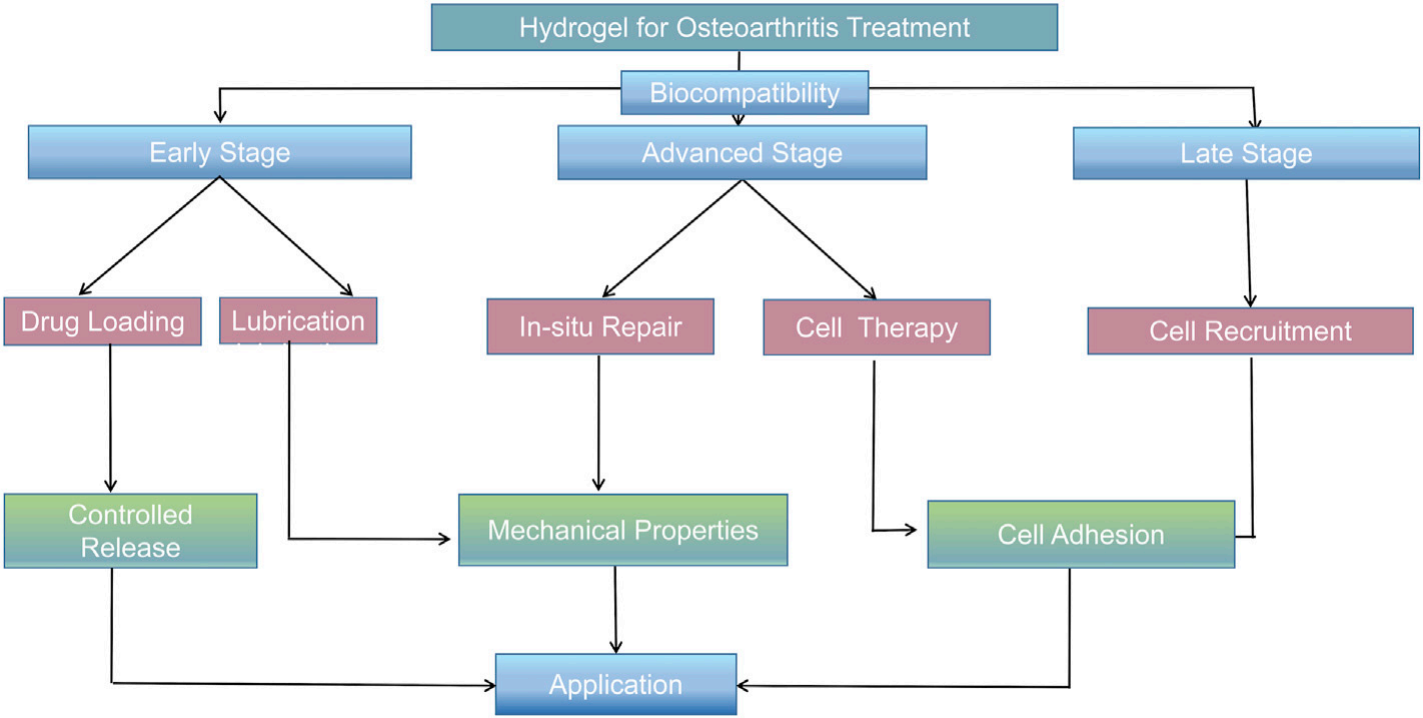
Hydrogel logic diagrams designed for different types of osteoarthritis.

We review the application of hydrogel scaffolds in the treatment of osteoarthritis in the recent 3 years. They can be divided into natural, synthetic, and hybrid polymer scaffolds. They have their own advantages and disadvantages; hence, we put forward some suggestions for later research and development: 1) Because the joint cavity exists in a wet environment, although the current gelatin, collagen, and other protein hydrogel products show excellent adhesion, their adhesion performance in a wet environment still must be tested. The mimicking mechanism of the mussel may provide a new possibility for wet adhesion. 2) Osteoarthritis drugs and hydrogels form complex polymers. The use of currently known steroidal anti-inflammatory drugs and numerous other drugs, including traditional Chinese medicine, curcumin, and berberine, proved to be effective in the treatment of osteoarthritis. Further research is required on these Chinese patent drugs. 3) The treatment processes of osteoarthritis may be accompanied by open or conservative treatments, sometimes even combined with arthroscopy, photodynamic, and other auxiliary treatment methods. How to design suitable hydrogel materials for different treatment methods is the follow-up research direction. We classify osteoarthritis into early osteoarthritis, advanced osteoarthritis, and late osteoarthritis according to the degrees of clinical development and provide different hydrogel design strategies for different stages of disease development (Figure 6). For example, early arthritis without cartilage breakage must mainly involve lubrication and anti-inflammation of the joint cavity. Osteoarthritis can only be treated by cytology at the end stage. Therefore, according to the development of osteoarthritis, the optimal treatment strategies and the most suitable scaffold materials must be the main directions for follow-up studies.

There are two main approaches to treat OA with hydrogels. The first is to use the different properties of the hydrogel itself to treat different osteoarthritis. Using its lubricating properties and fluidity, the hydrogel could be injected directly into the joint cavity to improve the friction of joint movement and relieve the state of osteoarthritis. Otherwise, one may use its adhesion and mechanical strength to construct artificial cartilage, which may be a hard lubricating hydrogel. Another approach is to use hydrogel as carriers of drugs or cells, which promotes bone and cartilage regeneration by loading cells or drugs into the material before treatment or recruiting corresponding cells at the lesion site after implantation. This requires a personalized design of hydrogel scaffold materials. This article reviews the application of hydrogel scaffolds for different osteoarthritis lesions and shows the broad prospects for the treatment of OA. Through the cooperation of cytology, material science, and pathophysiology, the reversion of OA or even a cure could be attainable.
