# Supplementary material for: Hydrogels for Treatment of Different Degrees of Osteoarthritis
Source: Front Bioeng Biotechnol. 2022 Jun 6;10:858656. doi: 10.3389/fbioe.2022.858656 (PMC9207401; doi:10.3389/fbioe.2022.858656)
Supplement: Supplementary file 1 [file Table1.docx]

**Table1:** Summary of tissue-engineered hydrogel for different degrees of osteoarthritis.

| **Hydrogel in early stage of osteoarthritis** | | | |
| --- | --- | --- | --- |
| **Name (Abbreviation)** | Ingredients | Effect | Reference |
| **Gellan Gum hydrogel** | Gellan Gum (GG); polyvinyl alcohol (PVA) | 1. Biocompatibility 2. Injectable 3. Enhanced lubrication performance. Can be used as a viscoelastic supplement | (Leone et al., 2020) |
| **HA/PA+HA/PM hydrogel** | Sodium hyaluronate (HA);  poly-2-acrylamide-2-methylpropanesulfonic acid sodium salt (PAMPS); poly-2-methacryloyloxyethyl phosphoryl choline (PMPC) | 1. Friction coefficient is low to the level of natural cartilage 2. Promote cartilage regeneration and inhibit inflammation in rat arthritis model | (Xie et al., 2021) |
| **Pluronic+HA hydrogels** | Poloxamers; hyaluronic acid; β-Lapachone | 1. Can be used as a viscoelastic supplement 2. Injectable 3. Controlled‐release | (Diaz-Rodriguez et al., 2021) |
| **HA-VS/SH-2-PEG** | HA, Divinyl sulfon (DVS), Dithiol functionalized poly(ethylene glycol)（SH-2PEG） | 1. Viscosity supplement 2. Inflammation suppression 3. Triamcinolone Acetonide load capacity 4. Injectable, adjustable viscoelastic, controllable degradation rate | (Cai et al., 2019) |
| **N-chitosan-ADH-HA-ALD** | N-car-boxyethyl chitosan (N-chitosan)； adipic acid dihydrazide (ADH)； hyaluronic acid–aldehyde (HA-ALD). | 1. Viscosity supplement 2. Self-healing properties 3. Inflammation suppression | (Mou et al., 2021) |
| **GelMA-AGA hydrogels** | Acryloyl glucosamine (AGA)；Methacrylated gelatin (GelMA) | 1. better biocompatibility 2. larger cell attachment 3. Promote hyaline cartilage growth | (Suo et al., 2020b) |
| **injectable semi-IPN hydrogel based**  **on gelatin and HA** | Hyaluronic acid (HA); gelatin; oxidized  dextran (Dex70-ox) | 1. Sustained drug release 2. Inflammation inhibiting | (García-Fernández et al., 2020) |
| **GelMA@DMA-MPC microspheres** | Dopamine (DMA);  methacrylate gelatin (GelMA);  poly sulfobetaine methacrylate (pSBMA) | 1. Enhanced lubrication 2. Sustained drug release 3. Inflammation inhibiting | (Han et al., 2021) |
| **MGS@DMA-SBMA** | Dopamine (DMA); poly sulfobetaine methacrylate (pSBMA) microfluidic gelatin methacrylate sphere (MGS) loaded with diclofenac sodium | 1. Enhanced lubrication 2. Sustained drug release 3. Inflammation inhibiting | (Yang et al., 2020b) |
| **EPA-gelatin hydrogels** | Eicosapentanoic acid (EPA)；gelatin hydrogel | 1. Inflammation suppression 2. Controlled‐release | (Tsubosaka et al., 2020) |
| **EGCG-HA/Gelatin hybrid hydrogel** | Epigallocatechin-3-gallate (EGCG); Hyaluronic acid (HA); gelatin | Control inflammation, scavenge ROS, and enhance cartilage regeneration | (Jin et al., 2020) |
| **peptide-cell-hydrogel** | APETx2; GelMA microsphere | Inflammation inhibiting and ECM metabolic balance is regulated | (Bian et al., 2021) |
| **PDA@Lipo@HAMA hydrogel** | Poly-dopamine; liposomes;  hyaluronic acid methacrylate; positively charged liposomes | Drug carried through the cartilage matrix and releases under the stimulation of ROS. The apoptosis rate of chondrocytes decreases significantly under oxidative stress | (Lin et al., 2021a) |

| **Hydrogel in advanced osteoarthritis** | | | |
| --- | --- | --- | --- |
| **Cell Therapy** | | | |
| **Name (Abbreviation)** | **Ingredients** | **Effect** | **Reference** |
| **Supramolecular gelatin hydrogels** | Gelatin；methacrylic anhydride；Acrylate β-cyclodextrin (Ac-β-CD) | Load kartogenin and protein TGF- β, hMBSCs；promote hyaline cartilage and subchondral bone regeneration | (Xu et al., 2019) |
| **3D-bioprinted pore-forming**  **GelMA hydrogel** | GelMA hydrogel | Improve compression ratio and injection capability, allow diffusion, migration, and biodegradation of loaded hMSC, enhance tissue formation in vivo | (Ying et al., 2020) |
| **Gel-EPL/PBA** | Gelatin methacrylamide (GelMA)；ε-poly-L-lysine (EPL)；glucose ； phenylboronic acids (PBA) | Significant stress relaxation；  improved chondrocyte viability and cartilage differentiation of stem cells | (Wang et al., 2021a) |
| **BMSC-laden injectable Col-HA hydrogel** | Collagen type I-tyramine (Col-TA)；hyaluronic acid-tyramine (HA-TA)；Hyaluronic acid | Provide a great microenvironment for BMSC growth and cartilage diﬀerentiation both in vitro and in vivo | (Zhang et al., 2020) |
| **supramolecular HA hydrogels** | β-cyclodextrin (β-CD)；adamantane (Ad); HA hydrogels | Encapsulate MSCs and improve chondrogenic diﬀerentiation and extracellular matrix deposition; Significant mechanical properties, such as shear thinning and self-repair | (Jeong et al., 2020) |
| **HA hydrogels modified with Foxy5 Peptide** | Foxy5 peptide; hyaluronic acid hydrogel | Enhance chondrogenesis and reduce hMSCs hypertrophy | (Deng et al., 2021) |
| **NC-CS/GP-21 hydrogel** | Glycerophosphate (GP); chitosan (CS); Nanocellulose (NC) | Biocompatible, injectable; mechanically stable;  and slowly degradable; the chondrogenesis ability of hDPSCs embedded in NC-CS/GP-21 hydrogel. | (Talaat et al., 2020) |
| **a combined triple interpenetrating network hydrogel** | Dextran; chitosan; teleostean | Load MSC to treat intervertebral disc degeneration | (Zhang et al., 2021a) |
| **GG/PEGDA DN hydrogel** | Gellan gum (GG); polyethylene glycol diacrylate (PEGDA) | Excellent mechanical and relaxation properties;  enhance chondrogenic diﬀerentiation | (Li et al., 2020) |
| **PEGS-OH hydrogels** | PEGS-  Az; PEGS-DBCO | Faster stress release due to incomplete network slip;  promote the adhesion and differentiation of bone mesenchymal stem cells | (Wu et al., 2021) |
| **Gelatin-SH/PEGDA IPN hydrogels** | (PEAD) polycation, heparin; insulin-like growth factor-1，thiolated gelatin (gelatin-SH)/poly(ethylene glycol) diacrylate (PEGDA) interpenetrating network (IPN) hydrogels | Bioactive IGF-1 is continuously released over three weeks; enhancing glycosaminoglycan deposition and  chondrogenesis-associated genes expression | (Cho et al., 2020) |
| **3D-bioprinted multilayer scaffold** | Methacrylated hyaluronic acid (MeHA); polycaprolactone; kartogenin; β-TCP | Help BMSCs survive and proliferate, and produce large amounts of cartilage-specific extracellular matrix | (Liu et al., 2021) |
| **CS hydrogel/3D-printed PCL hybrid scaffold** | Chitosan (CS); poly(ε-caprolactone) (PCL); TFNA | Promote proliferation and cartilage differentiation of SMSCs | (Li et al., 2021a) |
| **SAPH-coated PCL scaffolds** | Polycaprolactone (PCL), phenylalanine; glutamic acid; lysine | Significantly induce simultaneous regeneration of cartilage and subchondral bone after 8- and 12-weeks | (Li et al., 2019) |
| **Difunctional PEGS/MBG bilayer scaffold** | PEGylated poly(glycerol sebacate) (PEGS); mesoporous bioactive glass (MBG) | Reconstruct integrated articular hyaline cartilage and  subchondral bone in 12 weeks, exhibiting extraordinary regenerative efficiency | (Lin et al., 2020a) |
| **3D printed gradient hydrogel scaffold** | Nanohydroxyapatite; sodium alginate (SA) and acrylamide(AM); SA/AM hydrogel | Facilitate simultaneous regeneration of cartilage-subchondral bone | (Zhang et al., 2021c) |
| **Tri-layered stratified scaffold** | Gelatin methacrylamide (GelMA) hydrogel; zone-specific growth factor; melt electrowritten triblock polymer of poly(ε-caprolactone) and  poly(ethylene glycol) (PCEC) networks with depth-dependent fiber organization | Induce the MSCs to differentiate both the chondrogenic and osteogenic lineages | (Qiao et al., 2021) |
| **GG/ALG-based Ca-enriched acellular bilayer hydrogel** | Gellan gum; alginate; nHA | Imitate the mechanical properties and interface structure of osteochondral tissue; BMSC can be induced to differentiate into cartilage and bone cells | (Xing et al., 2021) |
| **Heterogenous bilayer hydrogel scaffold** | GelMA; acryloyl glucosamine (AGA); vinylphosphonic acid (VPA) | Promote chondrocytes to produce collagen type II and glycosaminoglycans and upregulate the expression of chondrogenesis-related genes | (Chen et al., 2021b) |
| **In-situ hydrogel repair** | | | |
| **Name (Abbreviation)** | **Ingredients** | **Effect** | **Reference** |
| **AHAMA hydrogels** | Hyaluronic acid hydrogel；aldehyde group；methacrylate (AHAMA) | Significantly improved durability and stability in humid environments and higher adhesion strength | (Chen et al., 2021a) |
| **3D-Printed Photocurable**  **Natural Hydrogel** | Gelatin ；hyaluronic acid；methacrylic anhydride；photoinitiator | Enhanced mechanical properties; prolonged degradation time. | (Xia et al., 2018) |
| **dual network hydrogel** | Nano-Fe3O4；flexible AAm binary polymer | High strength, high toughness, rapid self-recovery, and cell compatibility | (Gang, 2022) |
| **double layer hydrogel** | Hydrophilic polyanion PSPMA brush；polyzwitterion PSBMA brush | Low friction, high strength | (Rong et al., 2020) |
| **BC-PVA-PAMPS double network hydrogel** | Bacterial cellulose (BC) nanofiber network; poly(vinyl alcohol) (PVA); poly(2-acrylamido-2-methyl-1-propanesulfonic acid sodium salt) (PAMPS) | Equivalent tensile fatigue strength of cartilage; excellent candidate for replacing damaged cartilage | (Yang et al., 2020a) |
| **PVA/Col-II chondrohydrogels** | Spolyvinyl alcohol; fCol-II | Interconnection, porosity structure, moderate elastic modulus, and good biocompatibility | (Lan et al., 2020) |
| **(NIPAAm-co-AAm) DNs hydrogels** | N-Isopropylacrylamide (NIPAAm)；2-acrylamido-2-methylpropanesulfonic acid (AMPS)；acrylamide (AAm) | Parallel to the strength, modulus and hydration of the natural articular cartilage；lower coefficient of friction | (Means et al., 2019) |
| **PDA-CS-PAM hydrogel** | Polydopamine；chondroitin sulfate polyacrylamide； | Tissue adhesion and ultra-mechanical properties | (Han et al., 2018) |
| **hydrogels with different cell gradient densities** | Magnetically controlled methods；methyl acrylate hyaluronic acid-photoinitiator LAP | Mimic the structural characteristics of articular cartilage with a natural cell gradient | (Zlotnick et al., 2020) |

| **Cell recruitment hydrogel in end stage of osteoarthritis** | | | | |
| --- | --- | --- | --- | --- |
| **Name (Abbreviation)** | **Ingredients** | **Bioactive substances** |  | **Reference** |
| **HAMA@HepMA blend MGs** | Methacrylated hyaluronic acid; heparin | Platelet-derived growth factor-BB (PDGF-BB); transforming growth factor-beta3 (TGF-β3) | Recruit endogenous stem cells ；Promote chondrogenic differentiation | (Lei et al., 2021) |
| **cellular cartilage tissue engineering system** | Chitosan/silk fibroin hydrogels | Stromal cell-derived factor-1(SDF-1)；kartogenin (KGN) | Promote mesenchymal stem cell recruitment and cartilage differentiation | (Dong et al., 2021) |
| **exosome-loaded high adhesion hydrogel** | Cross-linking network of sodium alginate-dopamine chondroitin sulfate and regenerated silk fibroin (AD/CS/RSF) | Exosomes | Promote the migration and expansion of endogenous BMSCs, promote the proliferation and differentiation of BMSC | (Zhang et al., 2021b) |
| **Gel-nano-sEVs hydrogels** | Laponite nanoclay; Gelma hydrogel | Human umbilical cord mesenchymal stem  cells derived small extracellular vesicles (HUC-MSCs-sEVs) | Promote cartilage regeneration | (Hu et al., 2020) |
